# Robotic radical hysterectomy for cervical cancer: current trends and controversies

**DOI:** 10.7150/jca.99705

**Published:** 2024-08-13

**Authors:** Jeeyeon Kim, Ha Kyun Chang, Jiheum Paek, Hyeon Ji Park, Hye Yeon Moon

**Affiliations:** 1 Division of Gynecologic Oncology, Department of Obstetrics and Gynecology, Ajou University School of Medicine, Suwon, Republic of Korea.; 2 Department of Obstetrics and Gynecology, Ewha Womans University Seoul Hospital, The College of Medicine, Seoul, Republic of Korea.; 3 Department of Obstetrics and Gynecology, Korea University Ansan Hospital, Korea University School of Medicine, Ansan, Republic of Korea.

**Keywords:** cervical cancer, robotic surgery

## Abstract

Minimally invasive radical hysterectomy (MIRH) is widely performed as a treatment for early-stage cervical cancer. However, in 2018, a randomized controlled trial (RCT) called the Laparoscopic Approach to Cervical Cancer (LACC) trial showed that MIRH had poorer oncologic outcomes compared to laparotomy. Since then, several clinical studies have supported this finding, and most surgeons now perform MIRH with limited surgical indications. However, most of the reported studies evaluated laparoscopic radical hysterectomy rather than robotic radical hysterectomy (RRH). Robotic surgery has advantages for complex surgical procedures in the deep and narrow pelvic cavity in cervical cancer, making it necessary to evaluate the benefits and potential harms of RRH individually.

Based on this systematic review, RRH is a safe and effective alternative to abdominal approach for early-stage cervical cancer. RRH offers significant perioperative benefits, including reduced blood loss, shorter hospital stays, and fewer complications, without compromising oncologic outcomes such as overall survival and progression-free survival. Additionally, surgeons should aim to minimize tumor cell spillage into the peritoneal cavity by eliminating the use of uterine manipulators or vaginal colpotomy. Ongoing RCTs will reveal whether we can perform RRH without oncologic compromise in cervical cancer.

## Introduction

The role of minimally invasive surgery (MIS) in the primary management of early-stage cervical cancer is currently a topic of great controversy. This issue was triggered by the publication of the Laparoscopic Approach to Cervical Cancer (LACC) trial, a large randomized controlled trial (RCT) 1 and an epidemiologic study 2. Both studies reported inferior oncologic outcomes with minimally invasive radical hysterectomy (MIRH) compared to abdominal radical hysterectomy (ARH) in patients with cervical cancer (Table 1). As the first RCT on this topic, the LACC trial's findings must be given serious consideration. The unexpected results of the LACC trial have raised questions about the safety of MIS for cervical cancer. Now, gynecologic oncologists question whether MIS still has a place in the treatment of cervical cancer.

There were many criticisms of the LACC trial, one of which is that only 15.6% of the procedures in the MIS arm were performed with robotic radical hysterectomy (RRH). It has been argued that robotic surgery has unique properties that make it better suited for performing complex procedures in the deep and narrow pelvic cavity in cervical cancer with fewer complications. 3,4. In comparison, with laparoscopic radical hysterectomy (LRH), it has been suggested that a high caseload is required to master the technique and maintain skills 5,6. In the LACC trial, each center only contributed a small number of patients to the MIS group, leading to questions about the surgical quality 7.

The SUCCOR trial, a large European observational cohort study, comparing disease-free survival (DFS) for patients with stage IB1 cervical cancer undergoing ARH versus MIRH has been published 8. This retrospective study echoed the findings of the LACC trial, reporting an increased risk of recurrence and mortality with the MIRH compared to the ARH group. The SUCCOR trial also found that performing protective maneuvers during MIRH, such as avoidance of the use of uterine manipulators (UM) and protective vaginal closure, was associated with outcomes comparable to ARH. Like the LACC trial, RRH was underrepresented in the SUCCOR trial, accounting for only 21.5% of the MIRH group.

Therefore, it is necessary to evaluate the benefits and potential harms of RRH individually. In this article, we provide a comprehensive review of all the contentious and topical themes surrounding the use of RRH in the treatment of early-stage cervical cancer.

## Oncologic outcomes and controversies in the LACC trial

In gynecologic cancer surgery, a radical approach is required, and studies comparing the outcome of MIS and open surgery were conducted prior to evaluating the outcome of robotic surgery. These studies found no difference in survival and oncologic outcomes between MIS and open surgery in endometrial cancer 9,10. Similarly, in the LACC trial, the outcomes of MIS and laparotomy were compared for patients with early-stage cervical cancer. This trial aimed to demonstrate that MIRH is not inferior to ARH, and DFS rate, recurrence rate, and overall survival (OS) rate were compared for stage IA1 to IB1 1. Among a total of 631 patients, 319 were in the MIS group (of which 84.4% were in the laparoscopic group and 15.6% were in the robotic group), and 312 were in the ARH group. As a result of this trial, DFS, OS rate, and recurrence-free survival (RFS) were significantly lower in MIS than in open surgery, and the death rate was significantly higher in MIS. Similarly, a review of MIRH in cervical cancer was conducted 11. This review included 22,593 patients, and progression-free survival (PFS) showed a lower rate in the MIS approach compared to open surgery, but there was no significant difference in OS between the MIS and open approaches.

After the LACC trial, the incidence of MIRH in cervical cancer decreased from 50% to 15%, and ARH became the standard operation. However, as mentioned in the LACC trial, there were limitations, and many recent studies have reported on them 12-16. The standardization of MIRH procedure has not been properly established, and cancer spillage may occur during surgery due to tumor exposure, the use of UM, and direct handling of the uterine cervix 12. The use of UM can cause intra-abdominal tumor cell spreading, and when used, the prognosis is poorer compared to open surgery. As mentioned in the LACC trial, recurrence was high during intracorporeal colpotomy in the MIS group, suggesting the possibility of tumor cell spread due to insufflation gas 17. According to the SUCCOR study, patients who underwent MIS with the use of UM (n = 144) had a 4.5-year DFS rate of 73%, compared to 83% in patients who did not use UM (n = 106; p < 0.001). Additionally, patients who underwent MIS without UM had a similar probability of relapse to those who underwent open surgery (hazard ratio [HR]: 1.58; 95% confidence interval [CI]: 0.79-3.15; p = 0.20) 8. In contrast, a study by Nica et al. compared 115 patients who underwent MIS with UM to 109 who did not use UM and found that the use of UM was not a significant risk factor for worse RFS in early cervical cancer patients (HR: 0.4; 95% CI: 0.2-1.0; p = 0.05) 18. Multivariate analysis indicated that tumor size and parametrium involvement were independent risk factors for OS. Therefore, considering methods to radically remove the visible mass in cervical cancer and proceeding with surgery can be valuable, allowing for further study of OS and PFS. Another limitation is that the operator's learning curve is not reflected, and the surgeon's surgical experience and skill affect the success of the radical surgery. In the case of skilled surgeons, the results show that the learning curve, surgical technique, and content are important in reducing the recurrence rate, even in MIS surgery 19-21.

## Approaches for reducing tumor spillage in MIRH after the LACC trial

Although the reasons for the poor outcomes associated with MIRH in the LACC trial are not clear, intraoperative cancer cell spillage due to tumor exposure, the use of uterine manipulators, or direct handling of the uterine cervix may be contributing factors 12. There is concern that laparoscopic surgery for malignant tumors may result in peritoneal dissemination, including port-site metastases. In cervical cancer, colpotomy under CO_2_ pneumoperitoneum may also be a risk factor for locoregional recurrence 22,23. Therefore, the use of a surgical technique to prevent the spillage of cancer cells in MIRH may be necessary to ensure favorable outcomes, similar to those seen in open surgery.

To avoid tumor manipulation and contamination, the use of UM should be discouraged 24. Although there is no clear evidence on whether transcervical UM affects pathological factors or trigger peritoneal dissemination, the LACC trial recommended using UM in all patients who underwent MIRH, and the prognosis may have been different if they were not used 12. A large-population observational cohort study of early-stage endometrial cancer showed that using UM might affect the prognosis 25. It found that the DFS in the no-manipulator group was similar to that of the open surgery group (HR = 1.58; 95% CI = 0.79-3.15), and the DFS in the protective maneuver group was also equivalent to that of the open surgery group (HR = 0.63; 95% CI = 0.15-2.59). Moreover, the negative effect of the UM may be more prominent in higher risk patients with larger tumors and fragile cells that may be more disseminated by the manipulator's iatrogenic effect.

The effects of the surgical procedure must be considered, and protective maneuvers need to be adopted because intracorporeal colpotomy provides a poorer oncologic outcome than vaginal colpotomy 22. Kanao et al. introduced the no-look no-touch technique during MIRH to avoid tumor spillage 26,27. These procedures are to create the vaginal cuff before MIRH and to avoid direct handling of the uterine cervix by sufficiently developing surgical spaces, including parametrial, paravesical, and pararectal spaces, and using a specimen bag when extracting it outside. Fusegi et al. compared the oncologic outcome of ARH and LRH using an above protective maneuver. There was no significant difference of the 3-year DFS rates (LRH 92.4% vs. ARH 94%) and OS rates between two surgical approaches. In addition, there was no significant difference of the 3-year DFS rates for patients with tumor sizes more than 2 cm between the two groups (LRH: 85% vs. ARH: 90.3%) 28.

## Studies regarding the oncologic outcomes of RRH

Because only 15.6% of patients in the MIS arm had undergone RRH in the LACC trial, some have suggested that the findings of the LACC trial do not apply to robotic-assisted surgeries 24. This concept should be carefully proven by high-quality prospective studies. Despite the increasing number of robotic surgeries in women with gynecologic diseases, oncologic outcomes have been scarcely reported. There are only retrospective and limited studies reporting comparable outcomes between RRH and ARH 29-33.

Sert et al evaluated 491 cervical cancer patients treated with RRH (n = 259) and ARH (n = 232) in a retrospective multi-center study. The authors showed that RRH had improved operative outcomes and comparable survival outcomes compared to ARH 29. The primary findings indicated that RRH was associated with reduced blood loss, shorter hospital stays, and fewer perioperative complications compared to ARH. However, no significant differences were observed in the OS (p = 0.48) and PFS (p = 1.00) during the mean follow up time of 39 months. The study concluded that RRH was a feasible and safe alternative to ARH, offering several perioperative benefits without compromising oncologic outcomes.

The Swedish group demonstrated that neither long-term survival nor the pattern of recurrence differed significantly between robotic and open surgery in a complete nationwide population-based cohort where RH for early-stage cervical cancer is highly centralized 30. This study evaluated the survival outcomes of 864 women with early-stage cervical cancer undergoing RRH (n = 628) versus ARH (n = 236). The study utilized data from national cancer registries and included a large cohort of patients. The results showed a 5-year OS rate of 94% for RRH and 92% for ARH, with PFS rates of 88% and 84%, respectively. When analyzing survival outcomes using propensity score matching with cohorts of 232 women each, no significant differences were observed. Both groups had a 5-year OS of 92%, with a HR of 1.00 (95% CI, 0.50-2.01). The DFS rates were 85% for RRH group and 84% for the ARH group (HR, 1.08; 95% CI, 0.66-1.78). Recurrence patterns did not differ significantly between the groups. Independent significant risk factors for DFS included tumor size (p < 0.001) and grade 3 tumors (p = 0.02). Additionally, the study highlighted that RRH was associated with lower intraoperative blood loss and shorter postoperative recovery times. The authors concluded that RRH is comparable to ARH in terms of survival outcomes and may provide improved perioperative benefits.

Another study was done by Shah et al. which also compared the surgical and oncologic outcomes of RRH (n = 109) and ARH (n = 202) in the treatment of early cervical cancer patients 31. The retrospective analysis included data from a single institution and examined variables such as operative time, blood loss, complications, and survival outcomes. The findings revealed that RRH resulted in significantly less estimated blood loss (EBL, 105.9 vs. 482.6 mL, p < 0.001) and shorter hospital stays (42.7 vs. 112.6 hours, p < 0.001) than ARH. There were no significant differences in OS (p = 0.85) and PFS (p = 0.230) between the two surgical approaches. The study concluded that RRH offers perioperative advantages without compromising oncologic effectiveness, supporting its use as an alternative to ARH.

Zhang et al. performed a meta-analysis to evaluate the efficacy of RRH compared to ARH and LRH for cervical cancer 32. The meta-analysis included high-quality studies that met stringent inclusion criteria. In the comparison of RRH with LRH, the analysis revealed no significant differences in operation time, EBL, conversion rate, intraoperative or postoperative complications, length of hospital stay, or tumor recurrence. When comparing RRH to ARH, patients who underwent RRH experienced significantly less EBL (weighted mean difference [WMD] = -322.59 mL; 95% CI: -502.75 to -142.43, p < 0.01), a lower transfusion rate (odds ratio [OR] = 0.14; 95% CI: 0.06-0.34, p < 0.01), and a shorter hospital stay (WMD = -2.71 days; 95% CI: -3.74 to -1.68, p < 0.01). Additionally, there were no significant differences between RRH and LRH regarding operation time, intraoperative or postoperative complications, number of retrieved lymph nodes, and tumor recurrence.

MEMORY (MulticentEr study of Minimally invasive surgery versus Open Radical hYsterectomy) study was done by Leitao et al., which is a multicenter investigation comparing MIRH (n = 715), including RRH (n = 558, 78%), with ARH in early-stage cervical cancer management 33. The study aimed to assess survival outcomes, including OS and PFS, as well as perioperative complications and quality of life. The findings showed no significant differences in 3-year OS (95.8%, 95% CI: 93.6-97.2% vs. 96.6%, 95% CI: 93.8-98.2%, respectively) and PFS (87.9%, 95% CI: 84.9-90.4% vs. 89%, 95% CI: 84.9-92%, respectively) between MIRH and ARH. However, MIRH, including RRH, was associated with lower perioperative morbidity and faster recovery. The study highlighted that MIRH was a viable option for early-stage cervical cancer, providing comparable survival outcomes to ARH with additional perioperative benefits.

The summarized studies suggest that RRH is a safe and effective alternative to ARH for early-stage cervical cancer. RRH offers significant perioperative benefits, including reduced blood loss, shorter hospital stays, and fewer complications, without compromising oncologic outcomes such as OS and PFS. These findings support the use of RRH as a feasible option in the surgical management of early-stage cervical cancer, potentially improving patient recovery and quality of life while maintaining effective cancer control.

## Ongoing studies regarding robotic radical hysterectomy

There are currently several RCTs in progress, including the RACC (Robotic Approach to Cervical Cancer) and ROCC (Robotic versus Open Radical Hysterectomy for Early-Stage Cervical Cancer) trials (Table 2) 34,35. If the results of these RCTs are confirmed, they are thought to provide gynecologic oncologists with guidance regarding the role of robotic surgery in the treatment of early-stage cervical cancer.

The primary objective of the RACC trial is to investigate the oncologic safety of RRH for early-stage cervical cancer compared with standard ARH 34. The study's hypothesis is that RRH is non-inferior to laparotomy in terms of PFS, with the advantage of fewer post-operative complications and superior patient-reported outcomes. This trial is a prospective, multi-institutional, international, open-label RCT. Consecutive women with early-stage cervical cancer will be assessed for eligibility and subsequently randomized 1:1 to either RRH or ARH. Women over 18 years with cervical cancer stages IB1, IB2, and IIA1 squamous, adenocarcinoma, or adenosquamous will be included. The Primary endpoint is recurrence-free survival at 5 years between women who underwent RRH versus ARH for early-stage cervical cancer. Secondary outcomes include OS, perioperative morbidity, quality of life, diagnostic accuracy of the sentinel node algorithm, and healthcare costs. Data will be collected at multiple time points: pre-surgery, 1 month post-surgery, 6 months, 12 months, 24 months, and 60 months post-surgery. The trial is being conducted across various international centers, ensuring diverse participation and broad applicability of the results. The RACC trial employs specific procedural protocols for tumor containment during the robotic-assisted laparoscopic procedures. Intraoperative measures include documentation of operative time, intraoperative complications, blood loss, and use of sentinel lymph node biopsy. Postoperative measures assess hospital stay duration, recovery time, and patient-reported outcomes using validated questionnaires. Statistical analysis involves the Kaplan-Meier method for survival curves, the log-rank test for primary outcome comparison, and the Cox proportional hazards model for secondary outcomes.

The ROCC trial aims to overcome the limitations of the LACC trial by conducting a multicenter, prospective, randomized, non-inferiority trial 35. Its primary objective is to determine if RRH is not inferior to the open approach regarding 3-year DFS. Secondary objectives include disease-specific survival, OS, the patterns of recurrence, peri- and postoperative complications, long-term morbidity, impact on patient-reported outcome measures, and development of lower extremity lymphedema. Key inclusion criteria involve patients with stage IA2-IB2 adenocarcinoma, squamous cell, and adenosquamous cell carcinoma that have been histologically confirmed. The use of transcervical UM is prohibited intraoperatively, and specific detailed surgical techniques must be used for proper tumor containment. The ROCC trial employs rigorous intraoperative measures, including documentation of operative time, intraoperative complications, and blood loss, along with postoperative assessments such as hospital stay duration, recovery time, and patient-reported outcomes using validated questionnaires. Data will be collected at multiple time points: pre-surgery, 30 days post-surgery, 6 months, 1 year, and 3 years post-surgery. The ROCC trial also focuses on ensuring high standards of surgical technique and intraoperative management, with detailed recording of operative findings, intraoperative frozen sections for suspected metastases, and documentation of conversions to laparotomy. Statistical methods are similar to those in the RACC trial, with an additional emphasis on non-inferiority margins and confidence intervals for primary and secondary outcomes.

Both the RACC and ROCC trials are meticulously designed to provide robust evidence on the safety and efficacy of RRH in early-stage cervical cancer. The results of these trials are anticipated to have significant implications for surgical practice, potentially validating robotic surgery as a standard approach and offering enhanced outcomes for patients. By addressing both oncologic and quality-of-life measures, these studies aim to set new benchmarks in the surgical treatment of cervical cancer, guiding future clinical decision-making and patient care strategies.

## Conclusion

We have summarized current trends and controversies regarding RRH in the treatment of early-stage cervical cancer. While the LACC trial suggested that MIRH may be associated with poorer survival outcomes compared to ARH, further high-quality RCTs are necessary to confirm these findings. The results of these RCTs will provide much-needed clarity on the role of RRH in the management of early-stage cervical cancer. Until the results are reported, surgeons should aim to minimize the spillage of tumor cells into the peritoneal cavity by avoiding the use of UM or vaginal colpotomy. In addition, careful selection of patients for RRH in the management of early-stage cervical cancer is crucial.

## Author contributions

J.P. conceived the study. J.P. and H.K.C. designed literature search strategy and project implementation plan. J.K., H.K.C., J.P., H.J.P., and H.Y.M. conducted the literature search, wrote the manuscript. J.P. revised the manuscript and supervised this work. All authors reviewed and approved the final manuscript.

## Figures and Tables

**Table 1:** Two inferior results of MIS in early-stage cervical cancer

	Study period	Study design	Stage (FIGO 2008)	Control	Experimental	DFS rate	HR of recurrence (95% CI)	OS rate /4-year mortality	HR of death (95% CI)
LACC trial (NCT00614211)	2008-2017	Randomized, phase 3, multicenter trial	IA1 (lymphovascular invasion), IA2, or IB1 cervical cancer	312 to open surgery	319 to MIS	In MIS group, 94.3% In open group, 98.3%	HR 4.3; 95% CI, 1.4-12.6	In MIS group, 93.8% In open group, 99%	HR 6.0; 95% CI, 1.8-20.3
An epidemiologic study	2010-2013	National Cancer Database	IA2 or IB1 cervical cancer	1236 to open surgery	1225 to MIS	NR	NR	In MIS group, 9.1% In open group, 5.3%	HR 1.65; 95% CI, 1.22 - 2.22;

MIS, minimally invasive surgery; FIGO, international federation of gynecology and obstetrics; DFS, disease-free survival; HR, hazard ratio; CI, confidence interval; OS, overall survival; LACC, Laparoscopic Approach to Cervical Cancer; NR, not reported

**Table 2:** The baseline characteristics of RACC vs. ROCC Trial

Feature	RACC Trial	ROCC Trial (GOG-3043)
ClinicalTrials.gov Identifier	NCT03739944	NCT04831580
Start date	May 28, 2019	Mar 22, 2022
Objective	To compare RRH with open surgery for early-stage cervical cancer	To compare RRH with open surgery for early-stage cervical cancer
Primary outcome	PFS at 5 years	PFS at 3 years
Secondary outcomes	OS, perioperative morbidity, QOL, diagnostic accuracy of sentinel node algorithm, healthcare costs	OS, intraoperative and postoperative complications, QOL, healthcare costs
Study design	Multicenter, RCT, open-label	Multicenter, RCT, open-label, non-inferiority
Sample size	Approximately 800 participants	Approximately 800 participants
Eligibility criteria	FIGO stages IA2, IB1, IB2, no metastatic disease	FIGO stages IA2, IB1, IB2, no metastatic disease
Exclusion criteria	Neuroendocrine histology, history of pelvic or abdominal radiotherapy, other malignancies within 5 years	Neuroendocrine histology, history of pelvic or abdominal radiotherapy, other malignancies within 5 years

RACC, Robot-assisted Approach to Cervical Cancer; ROCC, Randomized Controlled Trial of Robotic versus Open Radical Hysterectomy for Cervical Cancer; GOG, Gynecologic Oncology Group; RRH, robotic radical hysterectomy; PFS, progression-free survival; OS, overall survival; QOL, quality of life; RCT, randomized controlled trial; FIGO, international federation of gynecology and obstetrics
